# Supplementary figures and images for: CaMKII controls neuromodulation via neuropeptide gene expression and axonal targeting of neuropeptide vesicles
Source: PLoS Biol. 2020 Aug 10;18(8):e3000826. doi: 10.1371/journal.pbio.3000826 (PMC7447270; doi:10.1371/journal.pbio.3000826)

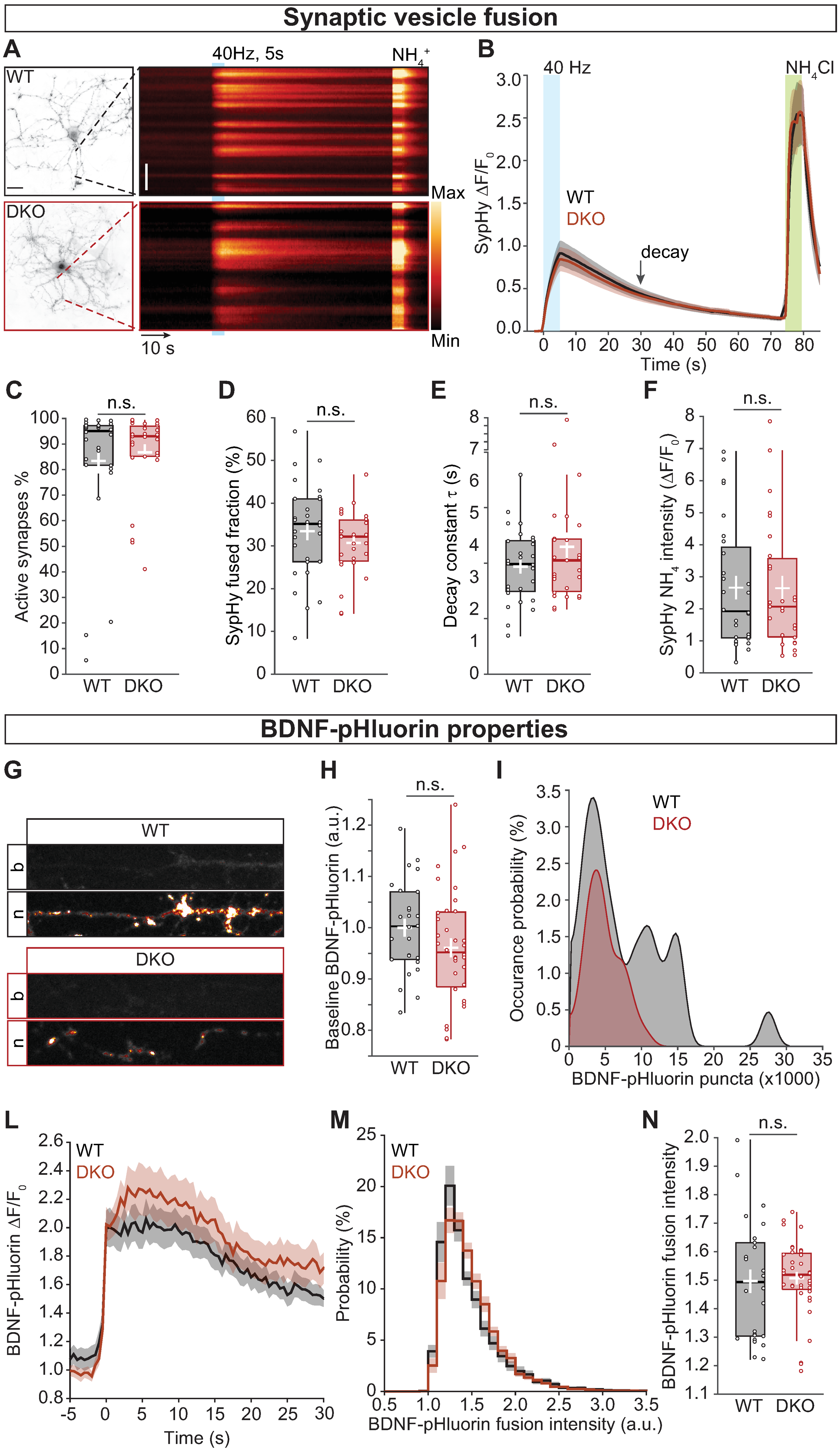

Supplement: S1 Fig — (A) Representative images of WT and DKO neurons infected with SypHy at the end of the electrical stimulation (left) and kymograph showing the dynamics of SypHy signal over time. (B) Average SypHy traces normalized as ΔF/F0. After 5 seconds of 40-Hz stimulation (light blue bar), neurons were allowed to rest for 60 seconds before NH4Cl superfusion to reveal the total amount of SypHy per synapses (light green bar). (C) Percentage of active synapses during a 40-Hz, 5-second stimulation. (D) SypHy fused fraction at the end of 5 seconds of 40-Hz stimulation. (E) Quantification of the decay constant τ for the SypHy signal intensity decay after stimulation. (F) Quantification of the SypHy signal upon NH4+ superfusion. (G) Typical neurite expressing BDNF-pHluorin during baseline, b, and during NH4+ superfusion, n. (H) Quantification of BDNF-pHluorin baseline fluorescence before stimulation. (I) Probability distribution of cells containing determined amount of BDNF-pHluorin positive puncta. (L) Average traces of BDNF-pHluorin fusion events aligned at the moment of fusion (0 seconds). (M) Histogram showing the BDNF-pHluorin signal intensity of individual fusion events. The fusion intensity was calculated as the fold change in fluorescence intensity from the 5 frames before fusion to the maximum intensity during fusion. (N) Quantification of average BDNF-pHluorin fusion intensity per cell. Traces show mean ± SEM (shaded area), boxplots with 95% CI whiskers, central bar is the median, white cross shows mean ± SEM. Columns and dots represent individual litters and neurons, respectively. The presented data can be found in S1 Data. *p < 0.05, **p < 0.01, ***p < 0.001. Scale bar = 25 μm (A images), 50 μm (A kymograph); kymograph color map in 1A, NanoJ-Orange in ImageJ. BDNF, brain-derived neurotrophic factor; CaMK, Ca2+/calmodulin-dependent kinase II; CI, confidence interval; SypHy, synaptophysin-pHluorinl; WT, wild type. (TIF) [file pbio.3000826.s004.tif]

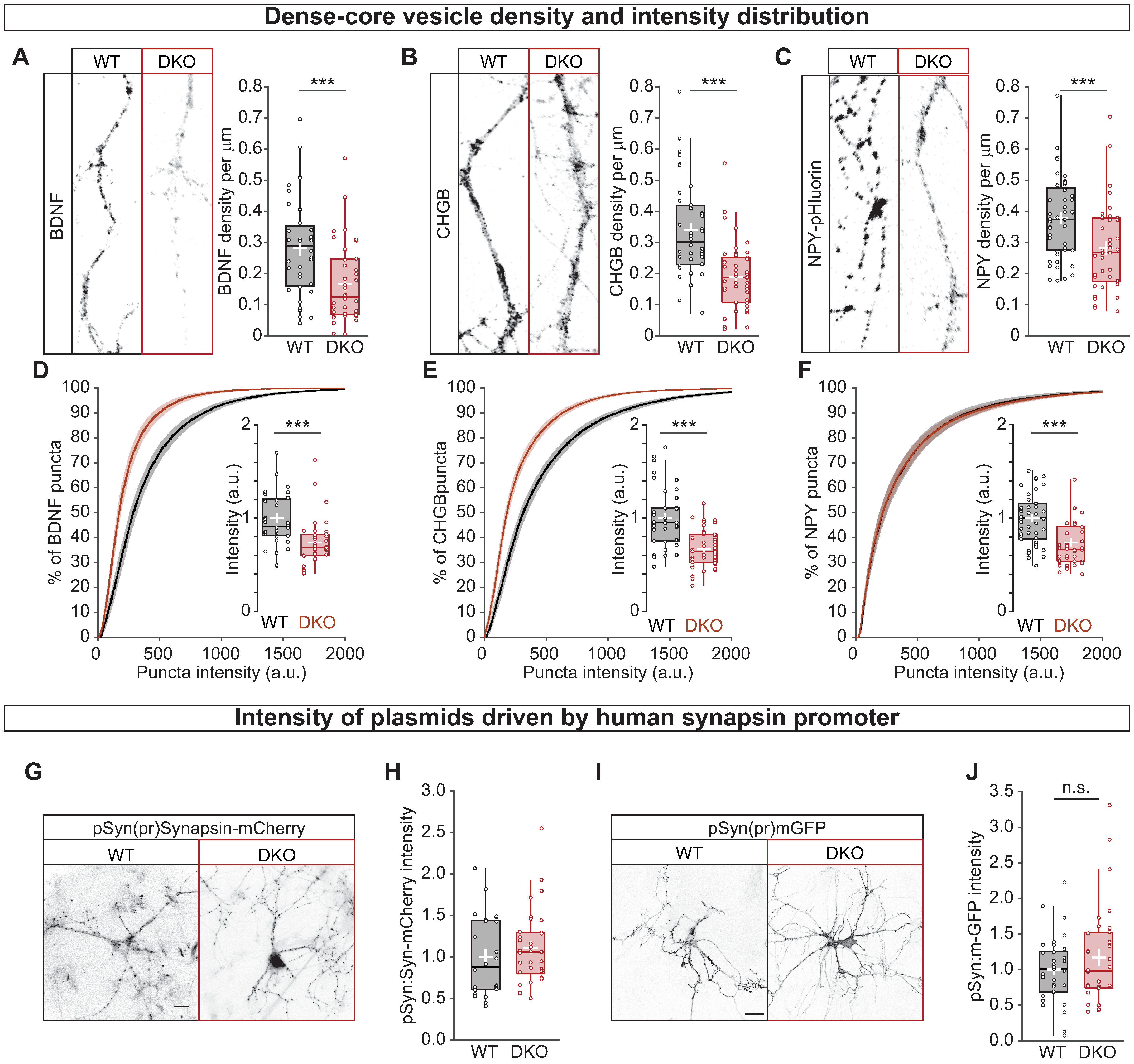

Supplement: S2 Fig — (A) Typical images of neurites of WT (left) and DKO (right) immunostained for BDNF. Right, quantification of BDNF intensity at VGLUT1 labeled synapses in WT and DKO neurons. (B) Typical images of neurites of WT (left) and DKO (right) immunostained for CHGB. Right, quantification of CHGB intensity at VGLUT1 labeled synapses in WT and DKO neurons. (C) Typical images of neurites of WT (left) and DKO (right) expressing NPY-pHluorin. Right, quantification of NPY-pHluorin intensity at VGLUT1-labeled synapses in WT and DKO neurons. (D) Cumulative probability and average intensity for single BDNF puncta. (E) Cumulative probability and average intensity for single CHGB puncta. (F) Cumulative probability and average intensity for single NPY-pHluorin puncta. (G) Typical neurons overexpressing Synapsin-mCherry (H) Quantification of the average intensity of Synapsin-mCherry per cell. (I) Typical neuron overexpressing mGFP. (J) Quantification of average mGFP intensity in the total neuritic arbor. Boxplots with 95% CI whiskers, white cross shows mean ± SEM, central bar is the median. Columns and dots represent individual litters and neurons, respectively. The presented data can be found in S1 Data. *p < 0.05, **p < 0.01, ***p < 0.001. Scale bar = 25 μm (G-I). BDNF, brain-derived neurotrophic factor; CaMKII, Ca2+/calmodulin-dependent kinase II; CHGB, chromogranin B; CI, confidence interval; DKO, double-knockout; mGFP, membrane-bound GFP; NPY, neuropeptide Y; SypHy, synaptophysin-pHluorin; VGLUT, vesicular glutamate transporter; WT, wild type. (TIF) [file pbio.3000826.s005.tif]

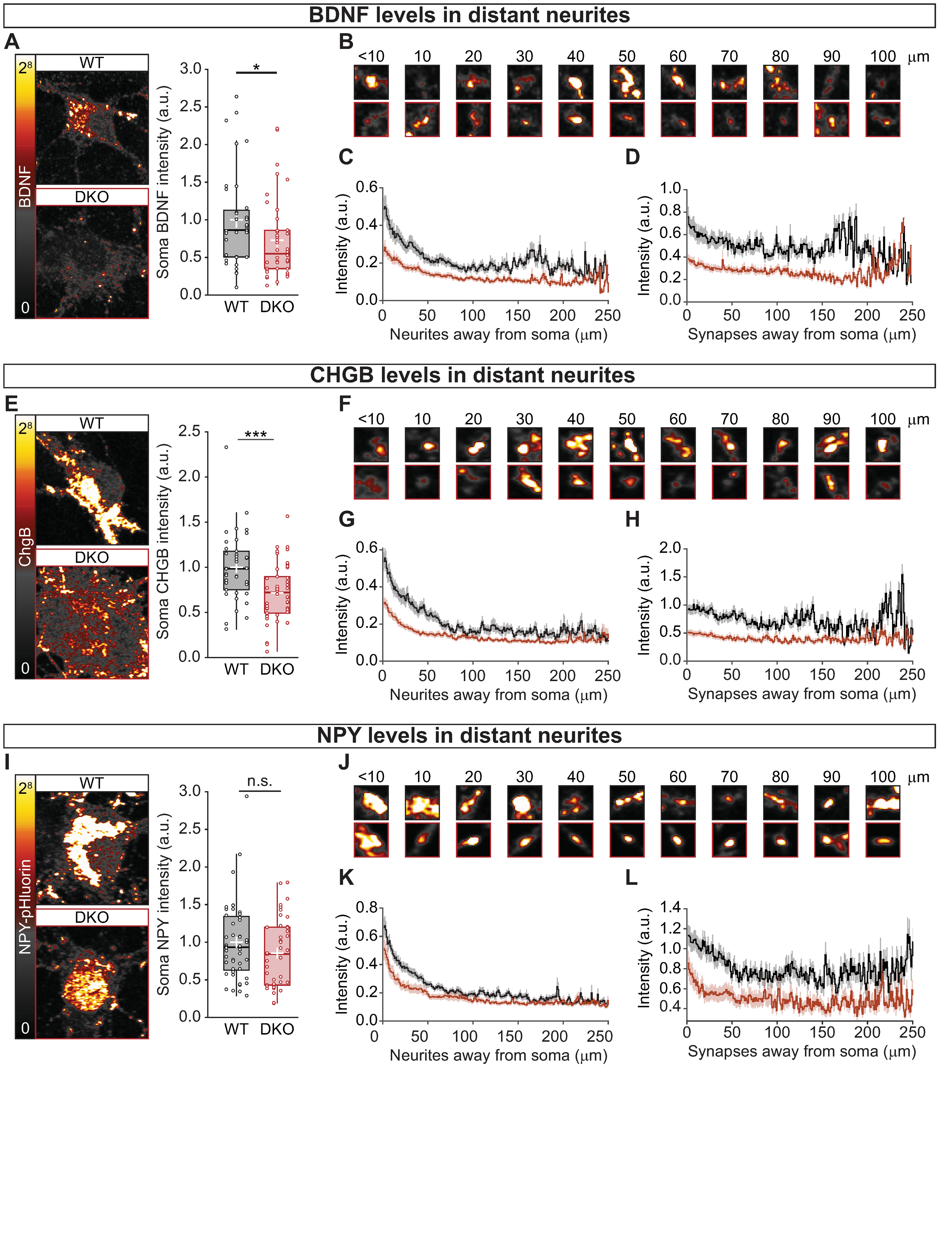

Supplement: S3 Fig — (A) Typical images of neurites of WT (top) and DKO (bottom) immunostained for BDNF. Right, quantification of BDNF intensity at the soma of WT and DKO neurons. (B) Typical synapses immunostained for BDNF at the indicated distances from the soma. (C) Intensity profile of BDNF immunoreactivity in correlation to the distance from the soma. (D) Intensity profile of BDNF in VGLUT1 positive synapses in correlation to the distance from the soma. (E) Typical images of neurites of WT (top) and DKO (bottom) immunostained for CHGB. Right, quantification of CHGB intensity at the soma of WT and DKO neurons. (F) Typical synapses immunostained for CHGB at the indicated distances from the soma. (G) Intensity profile of CHGB immunoreactivity in correlation to the distance from the soma. (H) Intensity profile of CHGB in VGLUT1 positive synapses in correlation to the distance from the soma. (I) Typical images of neurites of WT (top) and DKO (bottom) expressing NPY-pHluorin. Right, quantification of NPY-pHluorin intensity at the soma of WT and DKO neurons. (J) Typical synapses expressing NPY-pHluorin at the indicated distances from the soma. (K) Intensity profile of NPY-pHluorin immunoreactivity in correlation to the distance from the soma. (L) Intensity profile of NPY-pHluorin in VGLUT1 positive synapses in correlation to the distance from the soma. Boxplots with 95% CI whiskers, white cross shows mean ± SEM, central bar is the median. Columns and dots represent individual litters and neurons, respectively. The presented data can be found in S1 Data. *p < 0.05, **p < 0.01, ***p < 0.001. Full figure width = 36.46 μm (A-E-I) 4.25 μm (B-F-J). BDNF, brain-derived neurotrophic factor; CHGB, chromogranin B; CI, confidence interval; DKO, double-knockout; mGFP, membrane-bound GFP; NPY, neuropeptide Y; SypHy, synaptophysin-pHluorin; VGLUT, vesicular glutamate transporter; WT, wild type. (TIF) [file pbio.3000826.s006.tif]

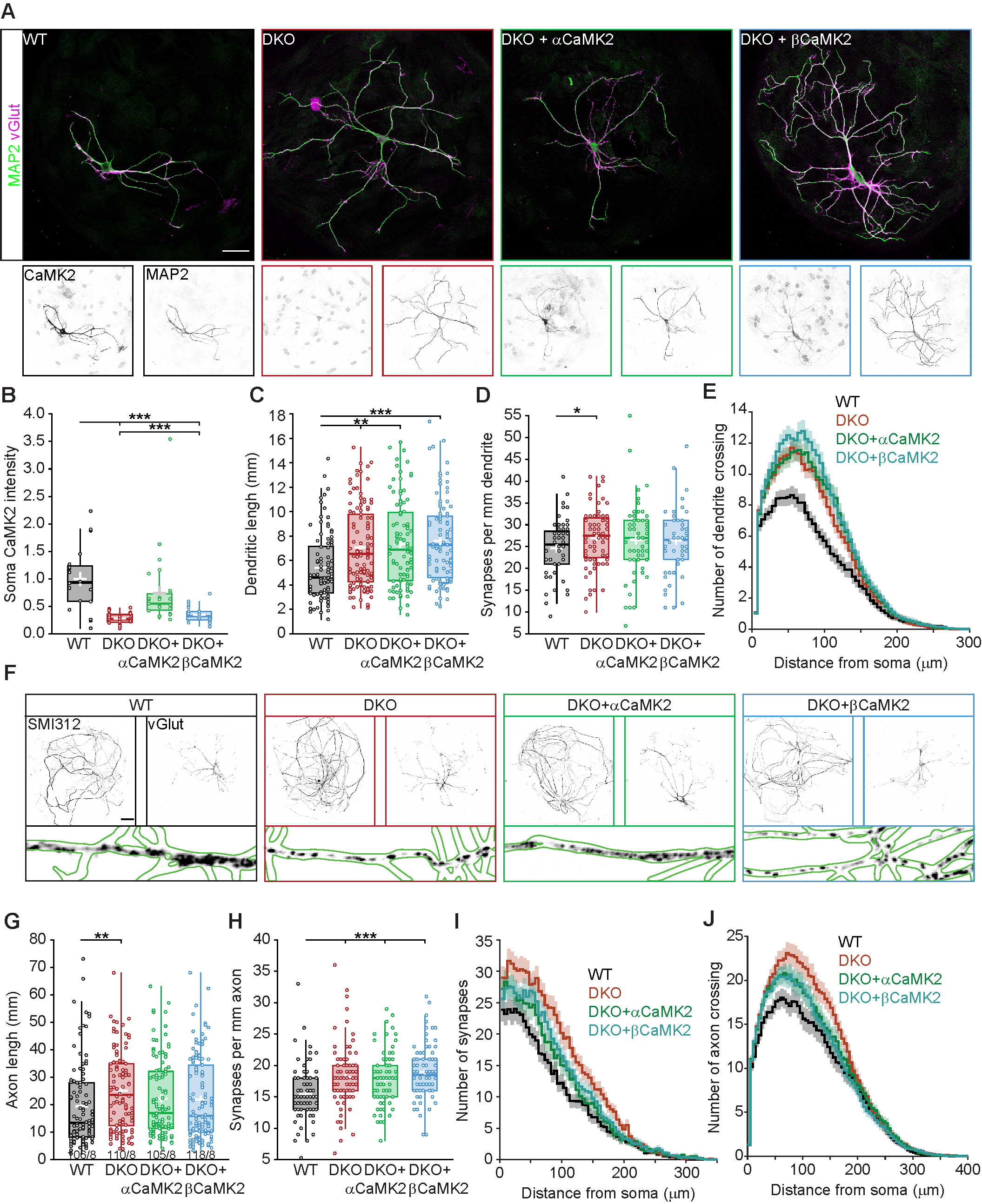

Supplement: S4 Fig — (A) Typical neurons grown on astrocyte micro-island immunostained for the dendritic marker MAP2 (green) and the synaptic vesicle marker VGLUT1 (magenta). In the black and white bottom insert, the same neurons immunostained for CaMKII. (B) Quantification of average somatic CaMKII intensity. (C) Quantification of average dendritic length. (D) Quantification of synapse density (number of synapses per mm of dendrite). (E) Sholl analysis for the distribution of dendrite crossings. (F) Typical neurons grown on astrocytes micro-islands immunostained for the axonal marker SMI312 and synaptic vesicle marker VGLUT1. In zooms (bottom), light green lines define axons, and the VGLUT1 signal is inverted. (B) Quantification of average axonal length per cell in mm. (C) Quantification of synapse density (number of synapses per mm of axon). (D) Sholl analysis of synapse localization. (F) Sholl analysis for the distribution of axonal crossings. Traces shows mean ± SEM (shaded area), boxplots with 95% CI whiskers, white cross shows mean ± SEM. Columns and dots represent individual litters and neurons, respectively. The presented data can be found in S1 Data. *p < 0.05, **p < 0.01, ***p < 0.001. Scale bar = 50 μm (A). CaMKII, Ca2+/calmodulin-dependent kinase II; MAP2, microtubule-associated protein 2; VGLUT, vesicular glutamate transporter; WT, wild type. (TIF) [file pbio.3000826.s007.tif]

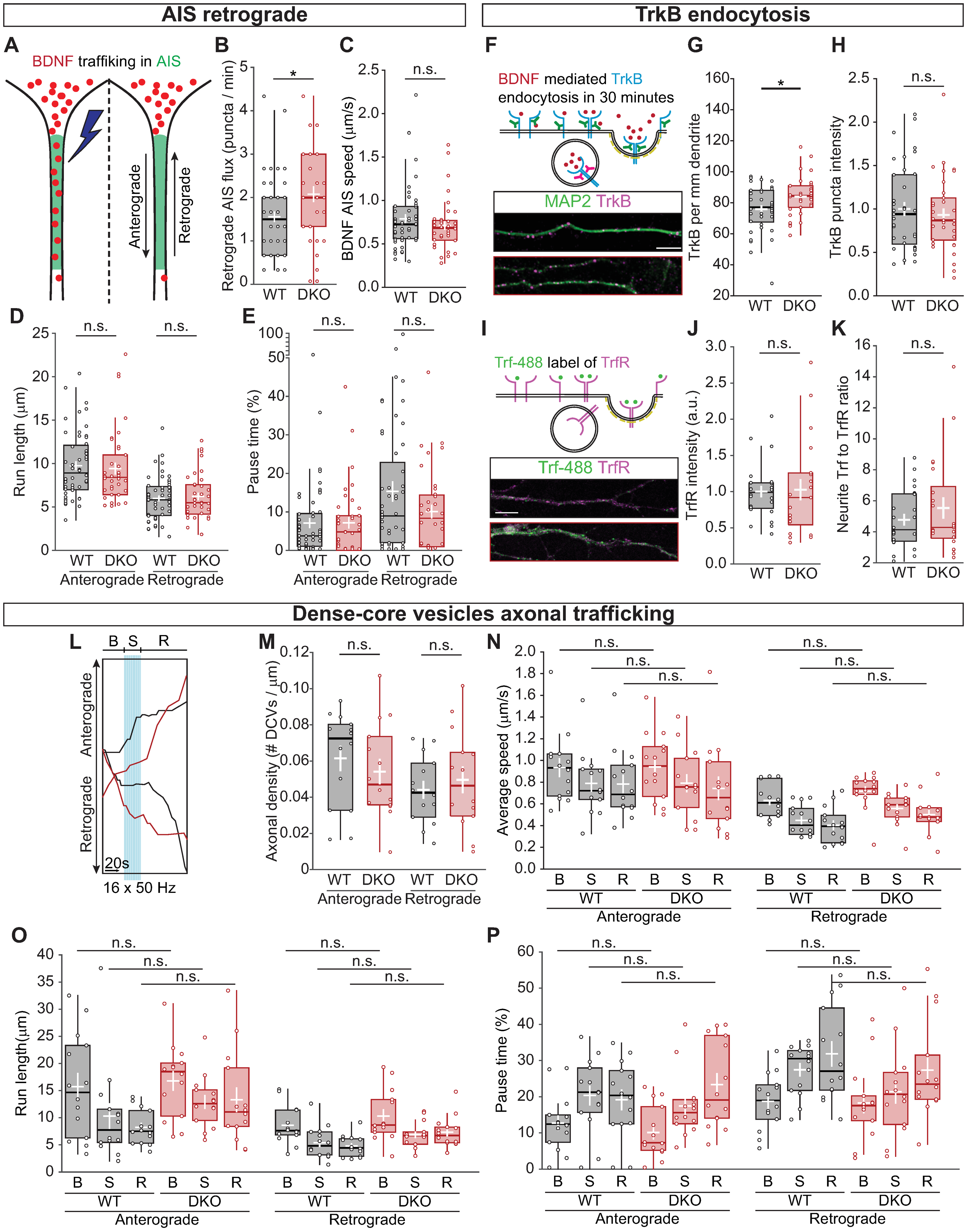

Supplement: S5 Fig — (A) Schematic representation of AIS targeting; the region of the AIS was visualized by NavII-III BFP. BDNF-mCherry vesicles were bleached at the AIS to allow quantification of new vesicles entering the axon. (B) Quantification of DCV flux at the AIS calculated as the number of BDNF-mCherry positive puncta that enter the NavII-III BFP area per minute in retrograde (from the axon to the soma) direction. (C) Quantification of BDNF-mCherry speed at the AIS in the retrograde direction. (D) Quantification of the run length in μm at the AIS in the anterograde (left) and retrograde (right) direction. (E) Quantification of the pausing time, as percentace of the total moving time, at the AIS in the anterograde (left) and retrograde (right) direction. (F) Schematic representation of the TrkB endocytosis assay. Neurons were incubated with TrkB antibody for 30 minutes at 37°C. After fixation and prior to permeabilization, cultures were incubated with saturating concentration of secondary antibody. Endocytosed TrkB was visualized using standard immunostaining procedures. Bottom, typical WT neurites immunostained for MAP2 (green) and endocytosed TrkB (magenta). (G) Quantification of the average number of TrkB puncta per mm of MAP2 positive neurites. (H) Quantification of the average intensity of TrkB puncta. (I) Schematic representation of the surface TrfR assay. Neurons were incubated with Trf-Alexa-488 (Trf-488) antibody for 5 minutes after blocking endocytosis. Total amount of TrfR was visualized using standard immunostaining procedures. Bottom, typical WT neurites immunostained for Trf-488 (green) and TrfR (magenta). (J) Quantification of the total TrfR levels. (K) Quantification of the ratio between Trf-488 intensity to TrfR intensity. (L) Schematic representation of NPY-mCherry puncta moving in the anterograde and the retrograde direction in kymographs, light blue bars represent the stimulation of 16 trains of 50 APs at 50 Hz. Trafficking parameters were calculated before st [file pbio.3000826.s008.tif]

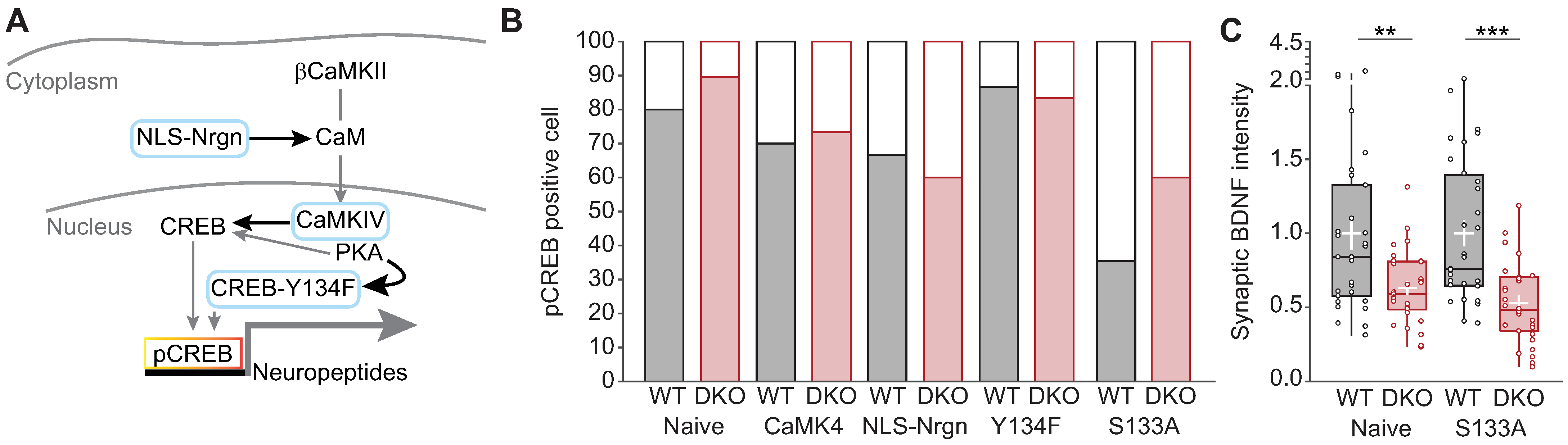

Supplement: S6 Fig — (A) Typical example of the downstream pathway of βCaMKII that lead to pCREB at Ser-133. CREB phosphorylation can be enhanced in the absence of CaMKII by shuttling CaM via NLS-Nrgn, by activating CaMKIV or by lowering the threshold for PKA-mediated CREB-Y134F. (B) Percentage of cells that presented pCREB in unstimulated conditions. (C) Quantification of the relative intensity of BDNF in synapses of WT and DKO neurons left untreated or with expression of the phosphodead form of CREB (CREB-S133A). Boxplots with 95 CI whiskers, white cross shows mean ± SEM. Columns and dots represent individual litters and neurons, respectively. The presented data can be found in S1 Data. *p < 0.05, **p < 0.01, ***p < 0.001. BDNF, brain-derived neurotrophic factor; CaM, calmodulin; CaMKII, Ca2+/calmodulin-dependent kinase II; CaMKIV, Ca2+/calmodulin-dependent kinase IV; CI, confidence interval; CREB, cAMP-response element binding protein; CREB-Y134F, phosphorylation of CREB with Tyr-to-Phe substitution at position 134; DKO, double-knockout; NLS-Nrgn, nuclear-localized neurogranin; pCREB, CREB phosphorylation; PKA, protein kinase A; WT, wild type. (TIF) [file pbio.3000826.s009.tif]

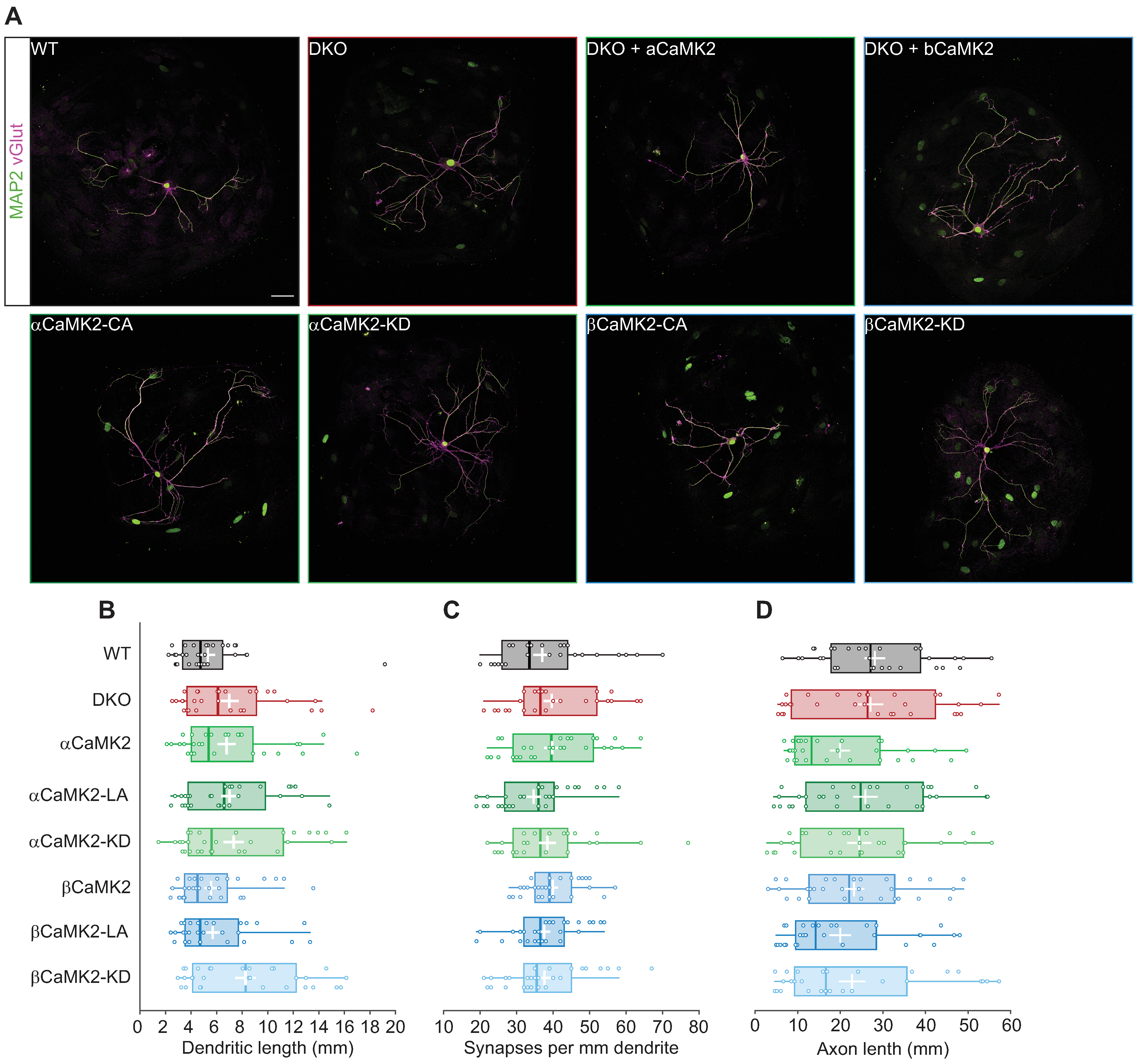

Supplement: S7 Fig — (A) Typical neurons grown on astrocyte micro-islands immunostained for the dendritic marker MAP2 (green) and the SV marker VGLUT1 (magenta). (B) Average dendritic length in mm. (C) Synapse distribution expressed in the number of synapses per mm of dendrite. (D) Average axonal length in mm. Boxplots with 95 CI whiskers, white cross shows mean ± SEM. Columns and dots represent individual litters and neurons, respectively. The presented data can be found in S1 Data. *p < 0.05, **p < 0.01, ***p < 0.001. Scale bar = 50 μm (A). CaMKII, Ca2+/calmodulin-dependent kinase II; CI, confidence interval; MAP2, microtubule associated protein 2; SV, synaptic vesicle; VGLUT1, vesicular glutamate transporter 1. (TIF) [file pbio.3000826.s010.tif]
