# Supplementary material for: CaMKII controls neuromodulation via neuropeptide gene expression and axonal targeting of neuropeptide vesicles
Source: PLoS Biol. 2020 Aug 10;18(8):e3000826. doi: 10.1371/journal.pbio.3000826 (PMC7447270; doi:10.1371/journal.pbio.3000826)
Supplement: S1 Raw Images — (PDF) [file pbio.3000826.s012.pdf]

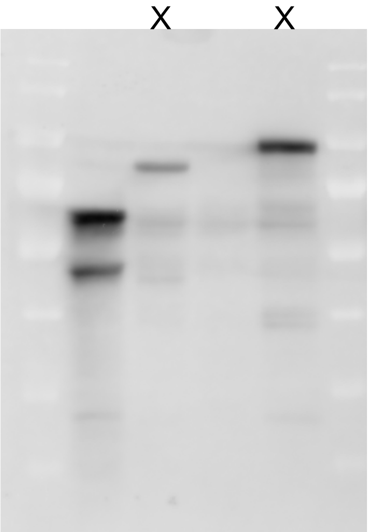

Raw image for Fig 1B top panel - CaMKII
Lane 1: Ladder
Lane 2: WT
Lane 3: CaMKII DKO + αCaMKII-CFP
Lane 4: CaMKII DKO
Land 5: CaMKII DKO + βCaMKII-CFP
Lane 6: Ladder
Alkaline phosphatase precipitation was used to visualize the protein of interest

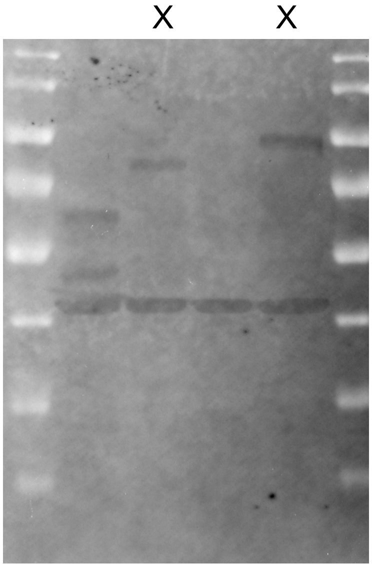

Raw image for Fig 1B low panel - Actin
Lane 1: Ladder
Lane 2: WT
Lane 3: CaMKII DKO + αCaMKII-CFP
Lane 4: CaMKII DKO
Land 5: CaMKII DKO + βCaMKII-CFP
Lane 6: Ladder
Alexa 488 was used to visualize the protein of interest

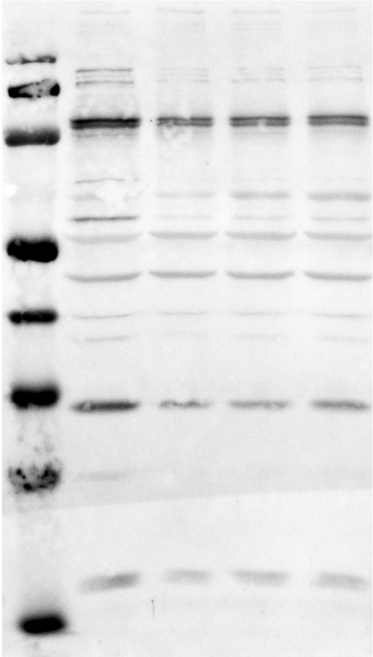

Raw image for Fig 5C top panel - Chromogranin B
Lane 1: Ladder
Lane 2: WT
Lane 3: CaMKII DKO
Lane 4: CaMKII DKO + αCaMKII-CFP
Land 5: CaMKII DKO + βCaMKII-CFP
Alkaline phosphatase precipitation was used to visualize the protein of interest

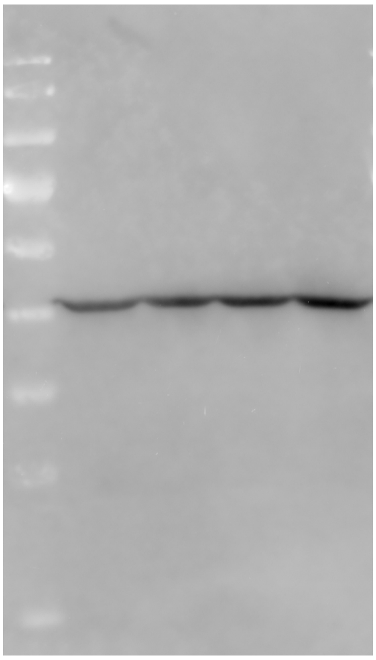

Raw image for Fig 5C top panel - Actin
Lane 1: Ladder
Lane 2: WT
Lane 3: CaMKII DKO
Lane 4: CaMKII DKO + αCaMKII-CFP
Land 5: CaMKII DKO + βCaMKII-CFP
Alexa 617 was used to visualize the protein of interest

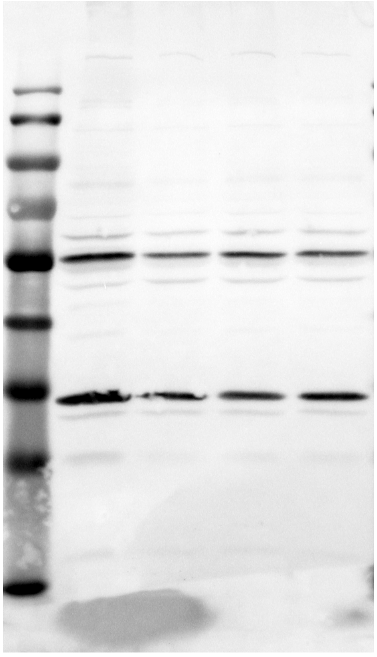

Raw image for Fig 5C low panel - Secretogranin II
Lane 1: Ladder
Lane 2: WT
Lane 3: CaMKII DKO
Lane 4: CaMKII DKO + αCaMKII-CFP
Land 5: CaMKII DKO + βCaMKII-CFP
Alkaline phosphatase precipitation was used to visualize the protein of interest

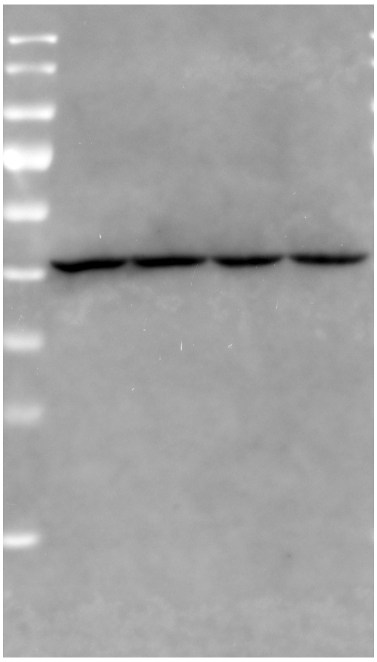

Raw image for Fig 5C low panel - Actin
Lane 1: Ladder
Lane 2: WT
Lane 3: CaMKII DKO
Lane 4: CaMKII DKO + αCaMKII-CFP
Land 5: CaMKII DKO + βCaMKII-CFP
Alexa 617 was used to visualize the protein of interest
