# Supplementary material for: CaMKII controls neuromodulation via neuropeptide gene expression and axonal targeting of neuropeptide vesicles
Source: PLoS Biol. 2020 Aug 10;18(8):e3000826. doi: 10.1371/journal.pbio.3000826 (PMC7447270; doi:10.1371/journal.pbio.3000826)
Supplement: Attachment — Submitted filename: Reply to the reviewersvPBIOLOGY-D-19-02844R1.docx [file pbio.3000826.s013.docx]

Revised PLOS Biology manuscript PBIOLOGY-D-19-02844R1 (Moro et al.)

REPLY TO REVIEWERS

We thank all three reviewers for the constructive comments. We are pleased that they found the manuscript “*provides clear evidence*” (reviewer 1), with a “*stringent approach*” (reviewer 2) providing “*novel and interesting findings*” (reviewer 3).

We have addressed their comments in an extensively revised version of the manuscript, adding six new data sets (Fig 7, Fig S2G-I, Fig S3A-F, Fig S4, Fig S7D-E, Fig S7I-K) to elucidate more of the mechanism how CaMKII regulates neuromodulator expression and fully comply with all other reviewer's requests. We also improved the analysis and better organized the manuscript. Please find below a point-by-point reply to the reviewers with their exact text in *blue/italic* and our response in black.

The new insights obtained on the mechanism of CaMKII-dependent regulation of DCV cargo expression also prompted us to make substantial adjustments to the abstract and discussion sections. We have removed some minor elements in favor of these new insights. All changes can be reviewed in the Word document with tracked changes.

Reviewer #1:

*This manuscript provides clear evidence that CaMKII in general and especially CaMKII beta is important for proper expression of cargo of dense core vesicles (DCV) including BDNF and chromogranins.
Experiments are well executed and conclusions justified (but limited).*

We thank the reviewer for the positive evaluation of our manuscript.

*Major Concerns:*

*1. My main concern is that the work is very focused and limited in scope. This manuscript would strongly profit if mechanistic insight into how CaMKII mediates its effect on cargo expression and secretory transport could be provided.*

**Reply:** We acknowledge the reviewer’s concern on the limited scope. We addressed this by conducting a series of experiments investigating the upstream and downstream signal cascades underlying the reduced neuromodulator expression in CaMKII DKO neurons (two new figures, 6 and 7, result section starts at line 360). In brief, we now demonstrate that secreted BDNF induces a positive feedback loop to increase proBDNF expression in WT neurons, but not in CaMKII-DKO, by blocking BDNF release with TeNT or scavenging secreted BDNF with antibodies (new Fig 6). Moreover, we have also elucidated the pathway downstream of CaMKII by restoring normal neuromodulator expression in CaMKII DKO neurons by expression of CaMKIV, NLS-Nrgn, or active-CREB (new Fig 7). The new Fig 8J is a schematic representation of the pathways.

*2. Fig. 2A: vesicular SEP-BDNF content was only quantified with NH4 after high frequency stimulation. That should be systematically done without such stimulation under basal conditions.*

**Reply:** We agree and have now also quantified the number of DCVs under basal conditions using the endogenous DCV-markers BDNF and ChgB, and exogenous DCV-maker NPY-SEP (new Fig S3A-F). These markers show the same reduction of DCV numbers in CaMKII DKO as our initial quantification of SEP-BDNF puncta with NH4. The released fraction of DCVs during stimulation is ~ 7% for both WT and DKO neurons. The number of fusion events is added to the estimation of the total number of DCVs in Fig 2A.

*3. Fig. 2B: it seems the SEP-BDNF puncta density is comparable for lower densities but the higher densities seen in WT neurons are where DKO neurons fail to match-perhaps this distribution can be further analyzed and interpreted. Is there a limit/ceiling effect of maximal capacity for DCV density?*

**Reply:** To answer the reviewer’s question, we calculated a probability density function (new Fig S2I) that shows a left shift in CaMKII DKO neurons with also a reduction at lower densities compared to WT neurons. A similar profile is found for the endogenous DCV markers BDNF and ChgB, and for NPY-SEP (new Fig S3A-F). We conclude there is no ceiling effect of the maximal capacity for DCV numbers other than neurite length (Persoon et al., 2018), which is not affected in CaMKII DKO neurons. We have added this conclusion to the revised manuscript in line 140

*4. The authors state: “ the minor changes in Syb2, Munc18, and SNAP25 suggest that CaMKII has a minor role…” (top of p9). A 30% reduction in Munc18 and 23% reduction in SNAP25 is not minor and could perhaps should?) affect vesicle release.*

**Reply:** We agree that the reduction in Munc18 and SNAP25 levels could have led to a defect in fusion efficiency. However, in our SypHy data (Fig S2A-F), we did not observe a reduction in the released SV fraction. Also, recent electrophysiological analysis (Kool et al., 2019) using the same CaMKII DKO mice did not reveal differences between WT and DKO. We changed the text to be more factual and better discuss our finding also in light of the new findings in (Kool et al., 2019).

*Minor Concerns:*

*1. BDNF is sometimes misspelled in Figures (Fig. 4B, left; Fig. 5B bottom BNDF2).*

**Reply:** We have corrected these typos and thank the reviewer for pointing this out.

Reviewer #2: Kang Shen - please note that this reviewer has waived anonymity

*Moro et al., studied the effect of CaMKIIs on dense core vesicle trafficking, release and neuropeptide expression in hippocampal neurons. They found that CaMKIIs increases the expression of multiple neuropeptide genes, most likely through regulation of transcription, an effect that is mostly carried out by the betaCaMKII. They also reported that the CaMKIIs increased the transport of DCVs into the axon but does not play a major effect on the release of DCVs. The strength of this manuscript lies in two folds. First they have taken the stringent genetic approach and examined the alpha and beta CaMKII double knockout cells. This clearly eliminated any specificity concerns from the pharmacology experiments and the rescue experiments demonstrated convincingly that the effects of these knockouts are due to CaMKIIs. Secondly, they have examined DCV release with single vesicle resolution which was not done in C. elegans.*

We thank the reviewer for the positive and in-depth evaluation of our manuscript and excellent suggestions.

*The weakness of the manuscript lies in the fact that the conceptual advance is rather limited. The regulation of CaMKII on BDNF transcription has been shown using drug experiments in mouse. The effect of CaMKII mutation in DCV abundance has been characterized in C. elegans. This manuscript confirmed these observations in mouse with a stringent experimental approach and showed that beta CaMKII is playing a more important role. However, the mechanisms of these effects are not explored. For example, examining the phosphorylation of CREB would be a potential way to know if the transcriptional effect is due to CREB. In the mammalian literature, CaMKIV has been more linked to CREB phosphorylation.*

**Reply:** We agree that the conceptual advance was somewhat limited in the original data set. This issue was also raised by reviewer 1. As indicated in the rebuttal to reviewer 1, point 1, we have now added two extensive new data sets elucidating both upstream and downstream signal cascades involved in CaMKII-dependent expression of neuromodulators. These new experiments also confirm the reviewer’s predictions that CaMKIV and CREB phosphorylation are part of the downstream cascade. The new data sets are presented in two new figures (Fig 6 and 7) and the new working model is in the new Fig 8J

*Below are my other technical comments.*

*In Fig. 1, is the fluorescence intensity before stimulation the same between wt and DKO?*

**Reply:** This is a valid point, and we added a new panel in Fig S2G-H, with the baseline fluorescence before stimulation, which is similar between WT and DKO.

*In Fig. 4, only the frequency of DCV movements was quantified. The authors should also quantify the speed and pauses of these movements. These information will further provide some insight in what is causing the reduced DCV entry into axon.*

**Reply:** We agree. We now include two new panels: Fig S7D showing DCV run length and Fig S7E reporting DCV pausing time. None of these parameters is altered in CaMKII DKO. Together, these data suggest that the reduced DCV entry into the axon is caused by less DCVs loaded onto kinesin motors without affecting kinesin transport dynamics.

*Line 143 “our data demonstrate that CaMKII regulates neuromodulation by lowering the
number of available DCVs and that is not involved in the fusion of SVs or the mechanism of fusion of DCVs in hippocampal neurons.” Shouldn’t this be increasing the number of DCV?*

**Reply:** Indeed, we conclude that CaMKII *increases* the number of available DCVs. We corrected this mistake and thank the reviewer for noticing this mistake.

*Line 258 “Therefore, CaMKII does not regulate the trafficking of DCVs in the axon but not take part in the 259 stimulation dependent arrest of trafficking vesicles.” This sentence is problematic. Shouldn’t it be “CaMKII does regulate…”?*

**Reply:** Yes, this sentence is problematic. We meant to say that CaMKII does not regulate trafficking properties, such as speed, pause time, and run length. We have now improved the text to explain this better. We thank the reviewer for pointing this out.

Reviewer #3:

*In this study, the authors investigated the necessity of alpha- and beta-CaMKII in the secretion of BDNF, a well-established neuromodulator in synapse development and plasticity using mouse alpha- and beta-CaMKII deficient, hippocampal cultured neurons. The findings were novel and interesting.*

We thank the reviewer for the positive evaluation of the manuscript.

*However, the manuscript in the current form requires substantial data to solidify sufficiency or necessity of either alpha- or beta-CaMKII in general neuromodulator secretion, given that most of findings were derived from exogenous overexpression of BDNF pHluorin only. In addition, the authors may want to show significance of their findings in conjunction with synapse development and/or plasticity. Therefore, the authors should perform experiments to test whether the loss of alpha- and beta- CaMKII affects- 1) synaptic vesicle secretion using vGlut- or synaptobrevin-pHluorin (or electrophysiological recordings), 2) exocytosis of general cargos using transferrin-receptor-pHluorin from dendrites. These experiments will confirm the authors’ claim on the regulatory role of alpha- and beta- CaMKII on neuromodulators.*
**Reply:** We agree it is important to also to assess possible roles of CaMKII on synaptic vesicle (SV) fusion and general (constitutive) cargos. As suggested by the reviewer, we have used a pHluorin-based reporter for SV fusion (synaptophysin-pHluorin (SypHy, (Granseth et al., 2006)). These data are presented in Fig S2A-F and from line 105. Our data shows that SV fusion is not affected in CaMKII DKO. Second, to address constitutively secreted proteins, we now include new data on surface labeling of endogenous TrkB and Trf-receptors (Fig S7F-K). Surface expression of both receptors is not affected in CaMKII DKO neurons. Together our data show that CaMKII is dispensible for DCV-, SV- or constitutive secretion.

*Minor points:*

*1. Is the number of BDNF puncta reduced in the distal dendrite of DKO neurons? Did the authors perform any detailed quantification of BDNF puncta in relation to their distances from the soma?*
**Reply:** We followed the reviewer’s suggestion and analyzed the intensity of endogenous BDNF, ChgB and exogenous NPY-pHluorin puncta in relation to their distance to the soma, both in neurites and in synapses. We observed that in CaMKII DKO neurons the signal intensity for all three markers was reduced throughout the neurite arbor, without major differences between proximal and distal synapses. The new data are presented in Fig S4 and from line 182.

*2. The authors may want to include recovery kinetics from FRAP experiments in Figure 4C and D.*
**Reply:** We did not describe this experiment clearly. The area corresponding to the axon initial segment was bleached to better quantify the number of DCVs that enter the axon per unit of time. Hence, it was not meant to test the fluorescence recovery after photobleaching. We have now added a better description of this experiments and also more in-depth quantification of the trafficking in Fig S7D, where we show DCV run length and S7E where we report DCV pausing time.

*3. BDNF was misspelled in multiple places (Figures 1G, 4B, and 5B).*

**Reply:** We have corrected these typos and thank the reviewer for pointing them out.

*4. Based on supplementary Fig 8, the overexpression of beta-CaMKII resulted in dramatic morphological changes. Why?*

**Reply:** This is a valid point. It was reported in rat cortical neurons in culture and hippocampal neurons *in vivo* that βCaMKII limits dendritic arborization via ubiquitin signaling to the centrosomes (Puram et al., 2011). Considering that we observed an increase in the total dendritic length mostly in DKO, DKO + αCaMKII and DKO + βCaMKII-KD, we conclude that this alteration is βCaMKII dependent as previously reported. We have now addressed this in the conclusion section line 201.

Additional references

Granseth, B., Odermatt, B., Royle, S.J., and Lagnado, L. (2006). Clathrin-mediated endocytosis is the dominant mechanism of vesicle retrieval at hippocampal synapses. Neuron *51*, 773-786.

Kool, M.J., Proietti Onori, M., Borgesius, N.Z., van de Bree, J.E., Elgersma-Hooisma, M., Nio, E., Bezstarosti, K., Buitendijk, G.H.S., Aghadavoud Jolfaei, M., Demmers, J.A.A.*, et al.* (2019). CAMK2-Dependent Signaling in Neurons Is Essential for Survival. J Neurosci *39*, 5424-5439.

Persoon, C.M., Moro, A., Nassal, J.P., Farina, M., Broeke, J.H., Arora, S., Dominguez, N., van Weering, J.R., Toonen, R.F., and Verhage, M. (2018). Pool size estimations for dense-core vesicles in mammalian CNS neurons. EMBO J *37*.

Puram, S.V., Kim, A.H., Ikeuchi, Y., Wilson-Grady, J.T., Merdes, A., Gygi, S.P., and Bonni, A. (2011). A CaMKIIbeta signaling pathway at the centrosome regulates dendrite patterning in the brain. Nat Neurosci *14*, 973-983.
